# Multiple Granular Cell Tumors of the New Born: A Case Report and Literature Review

**DOI:** 10.1002/ccr3.72034

**Published:** 2026-02-17

**Authors:** Fawzia M. Butt, Shamim A. Butt, M. L. Chindia

**Affiliations:** ^1^ University of Nairobi Nairobi Kenya

**Keywords:** congenital granular cell epulis, granular cell lesion, granular cell myoblastoma, Neumann tumor

## Abstract

Congenital granular cell epulis, a rare benign tumor, predominant in females present on the maxillary alveolar ridge. A newborn exhibited multiple oral tumors, interfering with breastfeeding. CT scan showed its confinement intra‐orally. The tumors were excised and surgical site closed primarily. Recovery was smooth with excellent prognosis.

## Graphical abstract

Preoperative clinical photo highlighting the intra oral maxillary masses.

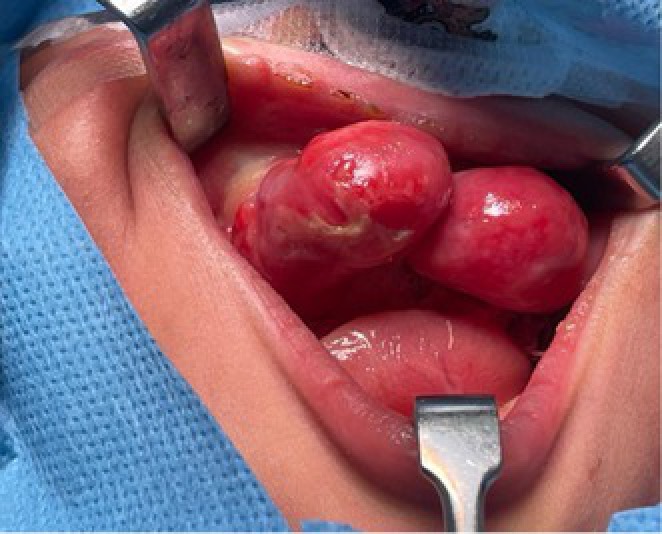

## 1 Introduction

In 1871, Dr. Franz Ernst Christian Neumann, a German pathologist, was credited for the description of an uncommon and unusual neonatal lesion termed the congenital granular cell epulis (CGCE). In Greek, Epulis means “swelling on the gingiva” [1]. Alternative nomenclature used for this lesion may include any of the following: Neumann tumor, granular cell myoblastoma, gingival granular cell tumor (GCT), granular cell fibroblastoma, Abrikosov tumor, granular cell lesion, and congenital epulis. However, the World Health Organization recommends the distinctive term “congenital granular cell epulis” [1, 2]. Approximately 250 cases of this benign lesion have been documented to date, with a female to male ratio of 10:1 [3, 4]. CGCE is a rare lesion and may be overlooked in the differential diagnosis of intraoral tumors [5].

Often, CGCE is present in the neonate's oral cavity as a soft lesion primarily in the alveolar ridge. It is a benign tumor with multiple lesions comprising nearly 10% of cases [3]. Though its histogenesis is unknown and debatable, it has been suggested that CGCE initiation emanates from undifferentiated mesenchymal, epithelial, nerve‐related cells, fibroblasts, pericytes, smooth muscle, or it could be neuroectodermal in origin [6]. Previous occasional cases of minor midface hypoplasia, an absent or a hypoplastic tooth have been reported. However, no cases are known to be associated with dental abnormalities or congenital anomalies [5]. Diagnosis is primarily clinical, making the differential diagnosis broad; hence, the importance of imaging cannot be overemphasized [4]. Tumor detection prenatally is possible via magnetic resonance imaging (MRI) or ultrasound (US) since CGCE manifestation occurs during the third trimester of pregnancy [3]. It is visible as a clear hypoechoic bulging oral mass accompanied by branched vascularization, with rapid growth during the third trimester of pregnancy and immediately ceasing at birth [2]. We present a case of CGCE noted immediately after birth.

## 2 Case History and Examination

**FIGURE 1:**
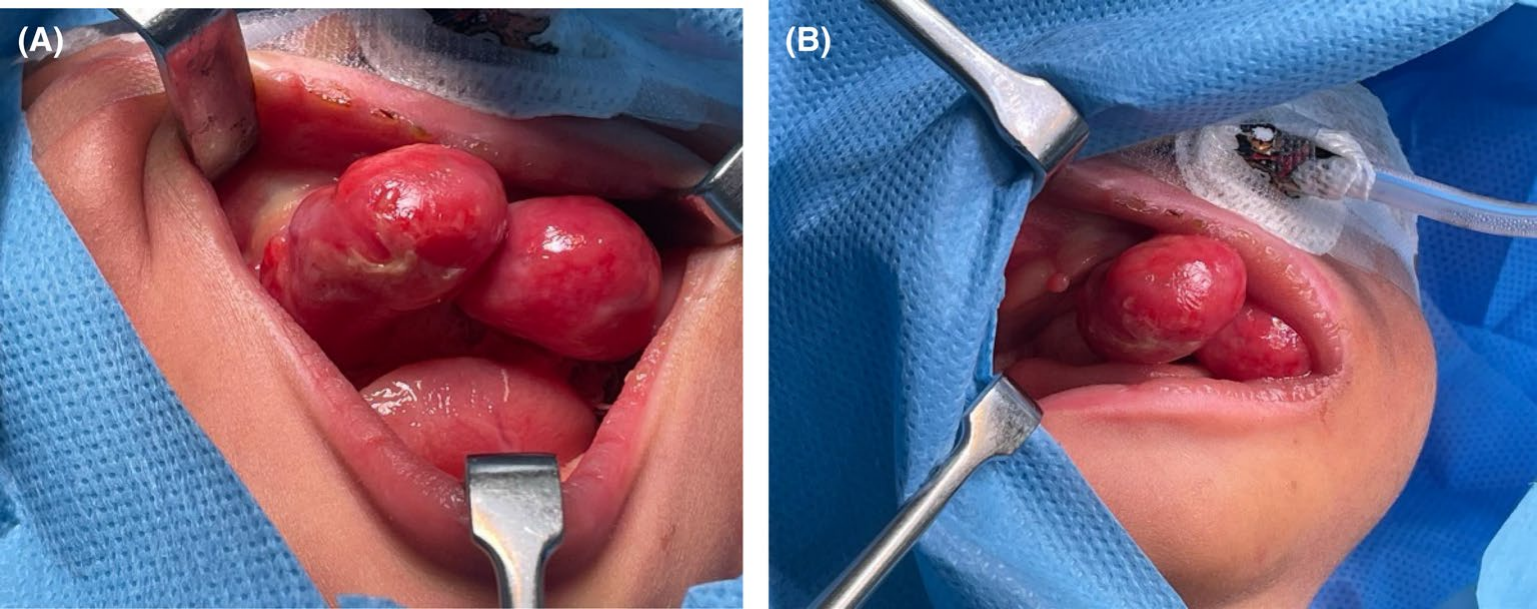
(A, B) Preoperative photo highlighting the exuberant pedunculate intra‐oral maxillary masses.

An Oral and Maxillofacial Surgeon was consulted one hour after the delivery of a female neonate weighing 3400 g via caesarean section, to assess multiple pink, pedunculated swellings protruding from the patient's oral cavity (Figure 1). The general pediatric assessment indicated impending challenges with breastfeeding and respiration. Notably, there was evidence of lip incompetence, as well as dryness and crusting. Intravenous support had been instituted for nutrition and hydration. Intraoral inspection revealed two large pedunculated masses {2.5 cm largest diameter on the anterior maxillary alveolar mucosa and three smaller ones on the mandibular alveolar ridge (5–8 mm in diameter)}. The mucosa was well vascularized and pink in color.

## 3 Methods (Investigations, Differential Diagnosis and Treatment)

An MRI of the head and neck was requested to rule out the presence of feeder vessels close to the larger masses. The radiology scan reported two pedunculated masses iso dense on T1W1 and mixed signal intensity on T2W1 with post contrast enhancement were seen that appeared to arise from the alveolar aspect of the palate without erosion or cleft of the palate. The left mass was reported to be round and measured 13 mm in diameter, and the right measured 26mmx10mmx10mm{anteroposterior (AP)X transverse (TV)X craniocaudal (CC)}. The mass abutted the tongue with inferior and with slight posterior displacement. The airway was otherwise intact. All other structures including the neck and visualized vasculature were unremarkable. A differential diagnosis of a granular cell epulis, melanotic neuroectodermal tumor of infancy, infantile myofibroma, and teratoma was made.

**FIGURE 2:**
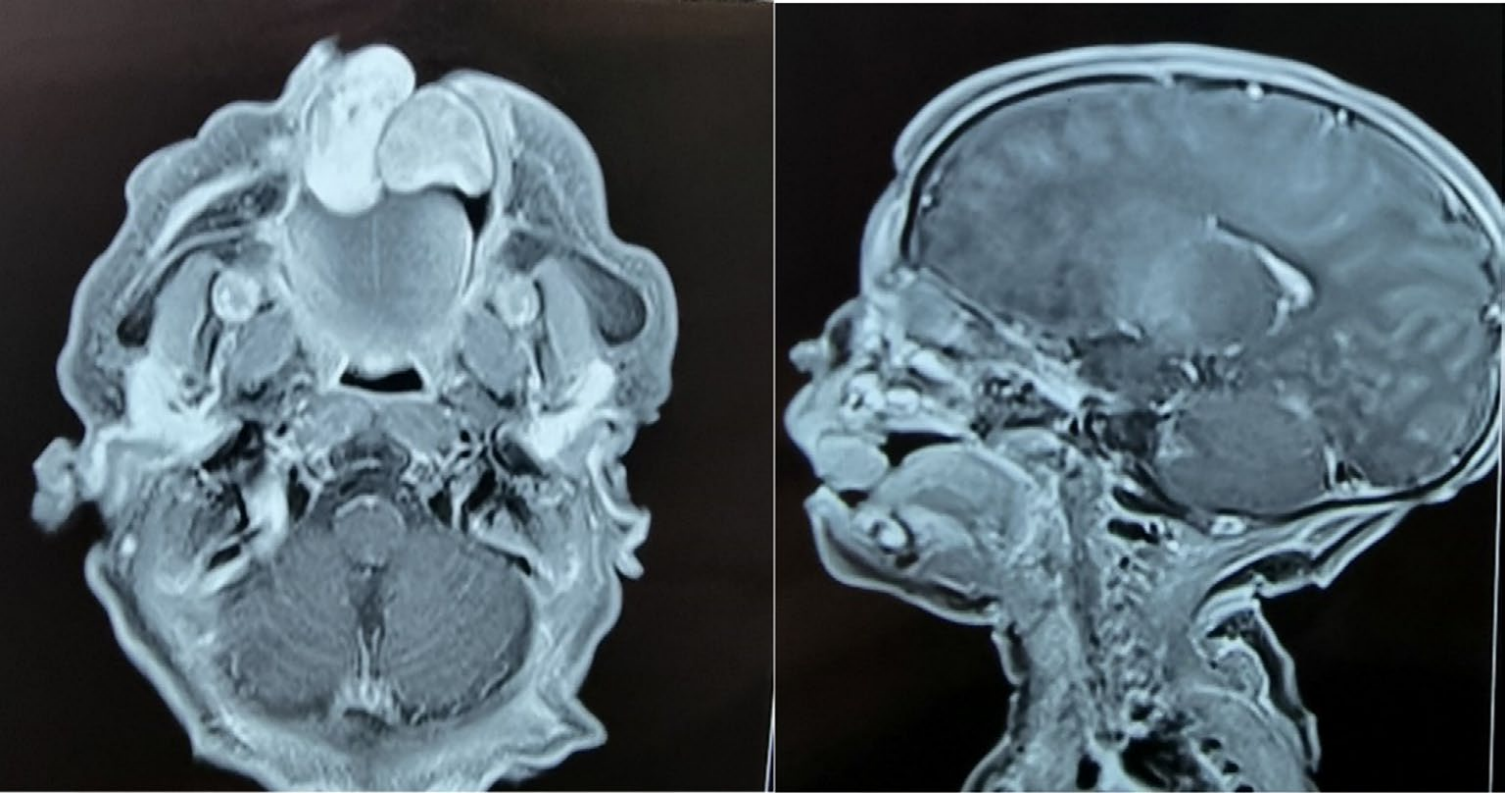
MRI showing two pedunculated masses which are iso dense on T1W1 and mixed signal intensity on T2W1 with post contrast enhancement seen that appear to arise from the alveolar aspect of the palate without erosion or cleft of the palate.

**FIGURE 3:**
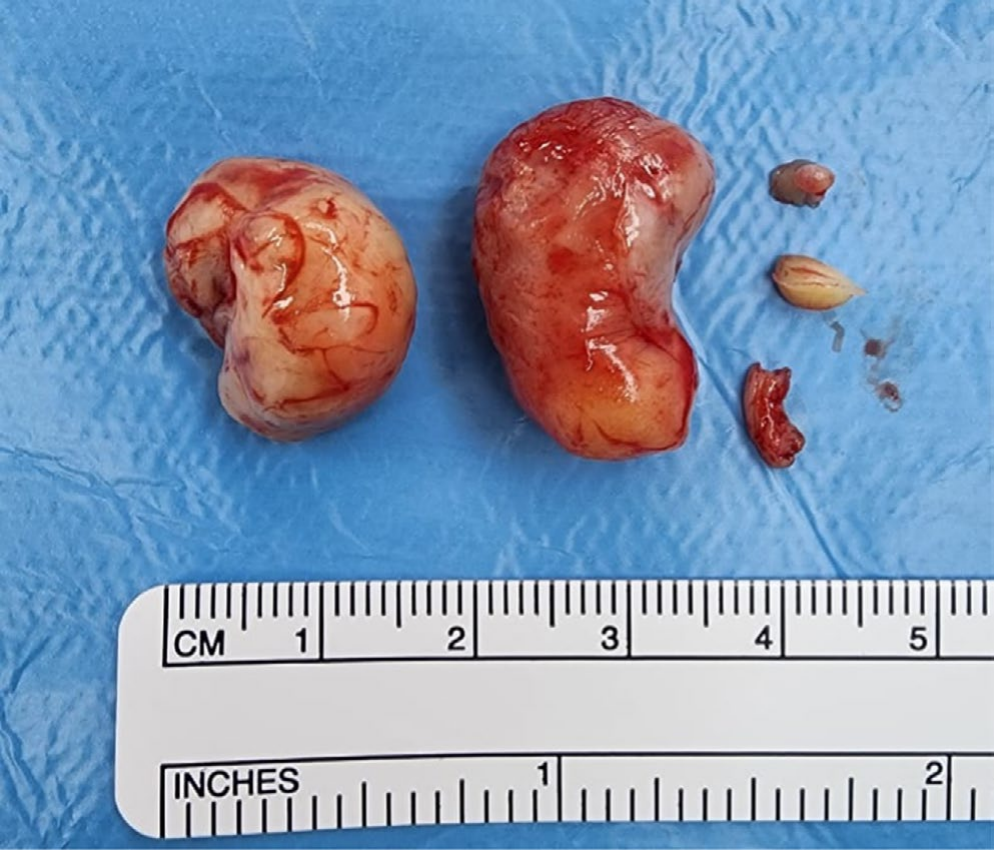
Intra operative photo of the five excised masses.

The radiological diagnosis was in keeping with the clinical differential diagnosis of a granular cell epulis (Figure 2). Theater was promptly scheduled because of feeding difficulties. Under general anesthesia using nasotracheal intubation, 0.5 mL of 2% lignocaine with adrenaline (1:80,000) was infiltrated around the tumor bases. The tumor was excised via an elliptical incision, and the margins were primarily closed with 6–0 vicryl sutures. The specimens were submitted for histopathology and immunohistochemistry (IHC) (Figure 3). The patient breastfed a few hours later and had an uneventful recovery. She was discharged on the third postoperative day with an excellent prognosis.

## 4 Results

**FIGURE 4:**
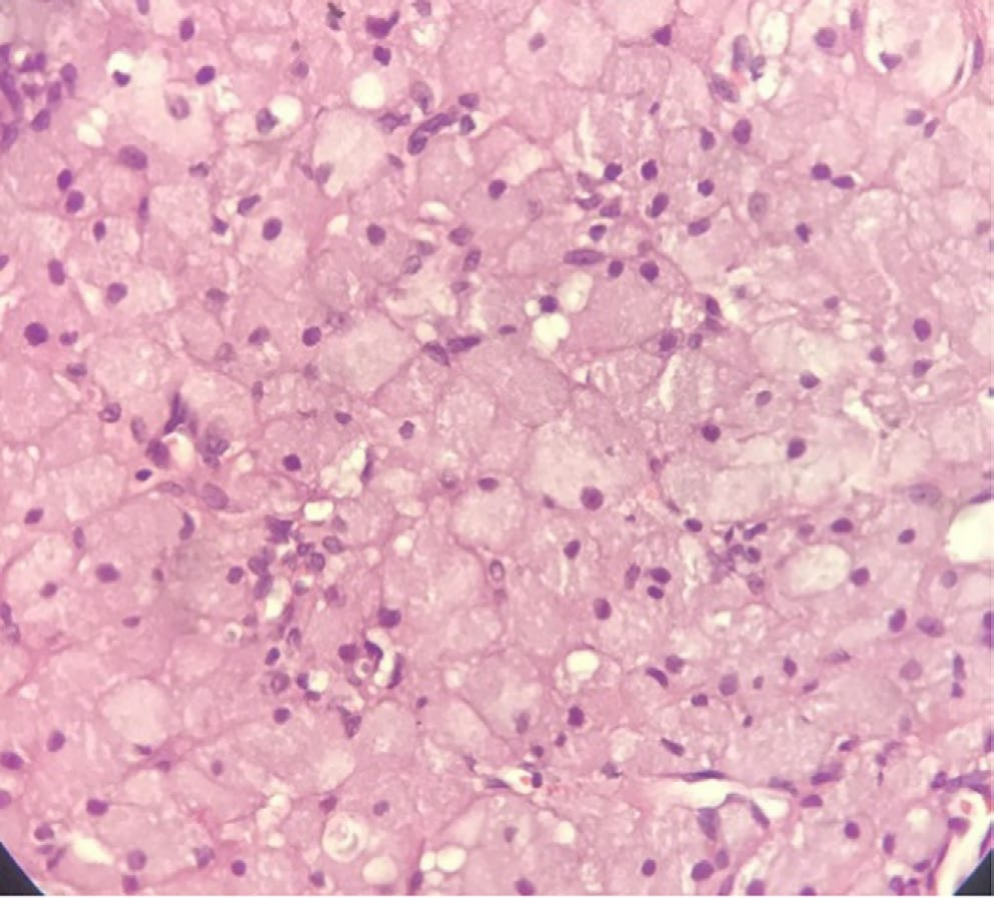
Hematoxylin and Eosin stain tissue (MagnificationX40) showing sections of a nodular tumor composed of sheets and nests of thin fibrous stroma. The tumor cells are rounded to polygonal cells with distinct cellular borders. There is abundant eosinophilic, granular cytoplasm, and smaller vesicular nuclei showing hardly any mitosis nor necrosis.

The histopathology report showed sections of a nodular tumor composed of sheets and nests of thin fibrous stroma. The tumor cells were rounded to polygonal cells with distinct cellular borders. There was abundant eosinophilic, granular cytoplasm, and smaller vesicular nuclei with no mitosis and necrosis. These features are consistent with those of CGCE (Figure 4.) The IHC report showed tumors cells were positive for Vimentin, neuron‐specific enolase (NSE), 8% positivity K167 for and negative for Desmin, Smooth Muscle Actin (SMA), (cytokeratin) CK, CD34, CD68, Inhibin, S100 and Calretinin.

## 5 Discussion

The occurrence of multifocal lesions within black neonates is found to be rare [7]. The last documented case in Kenya to our knowledge was reported in 1994 [8]. Owing to this rare occurrence, we report the third Kenyan case of a black female neonate who was postnatally diagnosed with CGCE [3]. CGCE tumors are typically soft, have a smooth surface, and are well‐defined. They may be pedunculated or sessile, multilobed, and display pink or red coloring. Their size can vary from just a few millimeters up to 9 cm in diameter. They are predominantly found more in the maxillary than the mandibular alveolus. Clinically, the lesion firmly attaches like a polyploid nodule to the labial or palatal gingiva. It can be erythematous or ulcerated, with the bone and teeth not involved [1, 2, 3, 5, 8]. Diagnosis can be based on clinical parameters; however, histopathological and histochemical criteria are equally fundamental in the determination of tumor diagnosis and management. The histological presentation was not of an atypical nature. The following IHC markers: S‐100, laminin, CD34, CD68, Nerve growth factor receptor (NGFR)/p75, inhibin‐alpha, chromogranin, desmin, keratins, SMA, CD31, and GLUT‐1 are absent during testing and those that test positive are Vimentin and NSE. As for our patient, the IHC tests showed the tumor to be negative for Desmin, SMA, CK, CD34, CD68, Inhibin, S100, Calretinin, while positive for Vimentin and NSE, identical to the trend reported [9, 10]. The proliferation index of the tumor disclosed was 8% showing Ki‐67 positivity which is similar to the range documented in some studies, that is, from 11.1%–16.7% and 15.1%–33.3% respectively [11, 12, 13].

**FIGURE 5:**
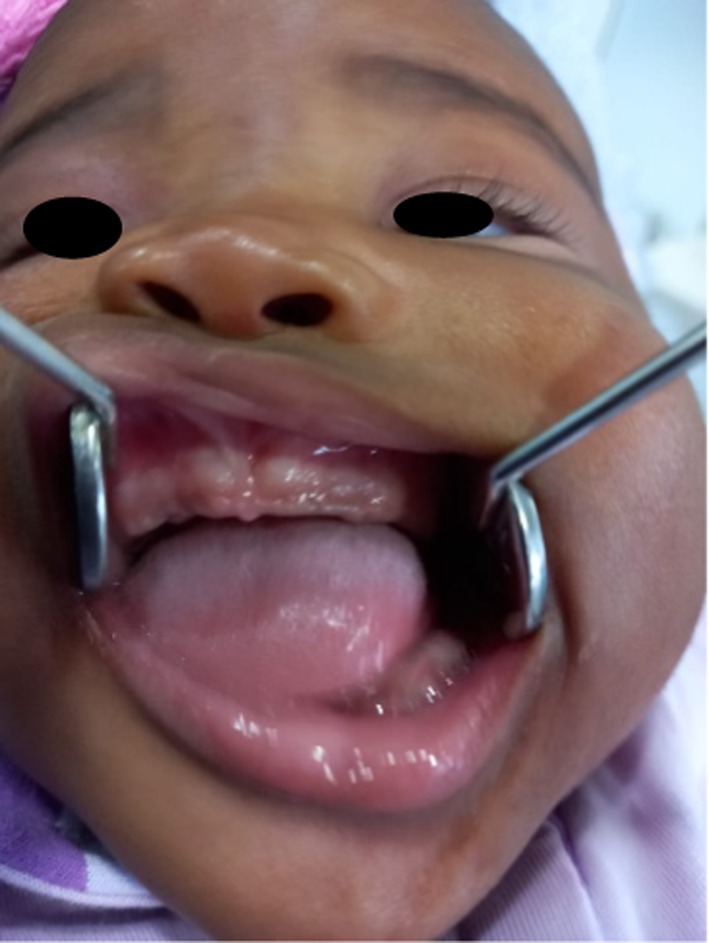
Post operative photo after a 6 month period.

The features outlined were typical of the tumor's description in the literature. The presence of multiple tumors, compounded by their size/s, may have led to the interference in the newborn's respiration and nutrition. Hence the need for urgent surgical intervention. The multidisciplinary team involved a gynecologist, neonatologist, maxillofacial surgeon, and anesthesiologist. The recommended treatment of choice is surgical excision, which usually has excellent prognosis [14]. Cases of recurrences have not been documented even when incomplete margins are excised, any malignant variation, or any futuristic disruption in teeth, nor gingiva [5]. There has been no association of CGCE with any syndromes or genetic defects; therefore, patients have no risk factors for other deformities or passing it on as an inherited gene [2]. After the meticulous excision we have had a follow up for a year and the patient has no signs recurrence (Figure 5).

## 6 Conclusion

Neonates born with CGCE need swift action due to the immediate respiratory and nutritional demands of the patient, especially if the tumors are multifocal and large in size. Although a rare and benign lesion, the surgeon must be familiar with the differential diagnosis to proceed with ease and diligence. The treatment plan for CGCE should embrace a multidisciplinary approach including counseling the parents/caregivers, reassuring them of treatment outcomes and life normalcy for the newborn as the review process continues to life normalcy.

## Author Contributions

**Fawzia M. Butt:** conceptualization, data curation. **Shamim A. Butt:** writing – original draft. **M. L. Chindia:** writing – review and editing.

## Funding

The authors have nothing to report.

## Consent

Written informed consent for this case report and associated photographs was obtained from the patient and has been uploaded.

## Conflicts of Interest

The authors declare no conflicts of interest.

## Data Availability

The data that support the findings of this study are available from the corresponding author upon reasonable request.
